# Domain adaptive uplift modeling across heterogeneous mental health cohorts

**DOI:** 10.1016/j.isci.2026.116027

**Published:** 2026-05-22

**Authors:** Abdullah Nayem Wasi Emran, A. B. M. Alim Al Islam

**Affiliations:** 1 Department of Computer Science and Engineering, Bangladesh University of Engineering and Technology (BUET), Dhaka, Bangladesh; 2 Department of Computer Science and Engineering, BRAC University, Dhaka, Bangladesh

**Keywords:** Neuroscience, Machine learning, Psychology

## Abstract

Predicting who will deteriorate under stress is important for targeting mental-health support; yet, treatment-effect models are rarely tested across populations. We evaluate a domain-adaptive neural uplift model on three heterogeneous cohorts—medical students, members of the general public under quarantine, and psychiatric patients (*n* = 2,624). The model combines a shared encoder, two potential-outcome heads, a domain discriminator, and an optional fairness penalty. We compare no-adaptation training with four domain-adaptation mechanisms under a leave-one-domain-out protocol, using AUUC and a semi-synthetic benchmark with known treatment effects. The model achieves positive uplift ranking in two cohorts, while the psychiatric cohort shows sign inversion of the effect proxy. Adaptation yields modest, tuning-sensitive gains over a strong baseline. These results clarify when domain adaptation helps treatment-effect ranking under distribution shift and inform cautious deployment across cohorts.

## Highlights

• Domain-adaptive uplift modeling transfers stress-risk rankings across cohorts
• No domain-adaptation method significantly outperforms a strong shared baseline
• Psychiatric fold failure traced to structural proxy-design leakage empirically
• Sigmoid adversarial weight schedules improve stability over fixed settings

## Graphical abstract

**Figure:**
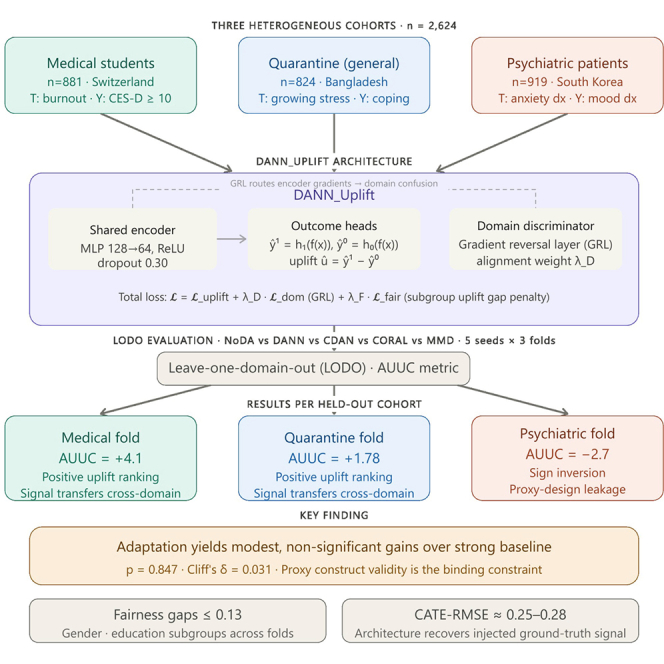

## Introduction

Mental-health deterioration under stress is clinically heterogeneous. Individuals exposed to similar stressors do not worsen to the same degree, and this variation reflects differences in vulnerability, prior symptoms, coping resources, and social context.^1^^,^^2^ This heterogeneity matters in practice because preventive mental-health resources are limited: clinicians and public-health systems often need to decide whom to monitor more closely, whom to refer, and who may benefit from low-intensity psychosocial or stress-management support.^3^ The deployment of machine learning in mental health holds immense promise for personalized care by enabling exactly this kind of individualized prioritization—particularly through uplift modeling, which seeks to identify which individuals show the greatest differential vulnerability under specific exposure conditions. In psychiatry and occupational health this capacity is foundational to the logic of *selective prevention*: the principle that limited clinical resources are most effectively directed toward individuals whose mental-health trajectory is genuinely modifiable by an available intervention.^4^^,^^5^

This clinical need motivates individualized prediction, but it also requires conceptual care. In the present study, we do not observe a single randomized psychiatric intervention shared across all datasets. Rather, we study a cross-dataset ranking problem using dataset-specific binary exposure or treatment proxies together with binary deterioration proxies. In the medical-student dataset, the exposure proxy is high burnout exhaustion (MBI exhaustion score at or above the 75th percentile^6^), and deterioration is operationalized as a CES-D score of ten or above.^7^ In the quarantine dataset, the exposure proxy is heightened self-reported perceived stress (binarized from yes/maybe vs. no), and deterioration is operationalized as self-reported coping failure—an established consequence of enforced social isolation.^8^^,^^9^ In the psychiatric dataset, the exposure proxy is an anxiety-spectrum diagnosis, and the adverse outcome is a comorbid mood disorder (MDD) or major depressive disorder.^10^ Accordingly, throughout this study, “treatment” refers to the dataset-specific exposure/intervention proxy and “deterioration under stress” refers to worsening under that proxy relative to its comparison condition. This framing supports a hypothesis about prioritized screening or support allocation; it should not be read as a direct claim that the estimated effects are already ready for clinical treatment recommendation.

This distinction is important because mental-health prediction models often fail to generalize cleanly across populations. Differences in measurement instruments, sampling frames, base rates, and contextual mechanisms can substantially change what a model learns and what its output means in a new setting.^11^^,^^12^ The same concern is relevant for our problem setting, where the three study populations differ not only in demographics and context, but also in how stress-related exposure and deterioration are operationalized.^13^ Furthermore, ensuring that these models remain fair across diverse demographic groups is essential to prevent the perpetuation of health disparities.^14^

Within this broader challenge, uplift modeling is attractive because it asks a more targeted question than standard risk prediction: rather than estimating who is generally likely to experience a poor outcome, it asks who appears differentially vulnerable under one condition relative to another—which is closer to the logic of targeted support and individualized intervention prioritization. Classical approaches to treatment-effect estimation—meta-learners such as the S-Learner, T-Learner, and X-Learner,^15^ and non-parametric methods such as causal forests^16^—offer flexible individual treatment-effect estimation within a single dataset. However, they do not natively support transfer across datasets with heterogeneous feature spaces and differing outcome distributions. Extending them to the multi-dataset setting requires either fitting independent models per dataset (discarding cross-population signal) or naively pooling samples with no mechanism to manage the resulting covariate shift—both unsatisfactory when the goal is generalization to a previously unseen study population. The neural architecture we adopt addresses both limitations simultaneously: a shared encoder learns representations across study populations, and the adversarial domain-confusion mechanism explicitly discourages encoding of dataset-specific artifacts that would impair out-of-dataset uplift ranking. Our no-adaptation (NoDA) variant, which disables the adversarial component, is functionally equivalent to a two-head neural T-Learner and thus serves as the direct neural counterpart to classical meta-learner baselines.

Mental-health datasets are rarely interchangeable, and this challenge is both empirical and clinical. Our recent cross-dataset mental-health study, which analyzed the same three study populations considered here from a descriptive and comparative perspective, showed that mental-health patterns vary meaningfully across medical, quarantine, and psychiatric settings.^13^ More broadly, Xu et al.’s GLOBEM benchmark demonstrated that longitudinal behavior models for depression detection often degrade markedly when deployed across datasets rather than within a single source population.^12^ This broader translational concern is consistent with psychiatric prediction research more generally, where systematic review evidence shows that generalizability and clinical utility remain major bottlenecks even when predictive performance within a development dataset appears promising.^11^ Clinically, long-standing diathesis-stress frameworks likewise suggest that stress exposure does not translate into deterioration uniformly across individuals; rather, outcomes depend on the interaction between stressors and pre-existing vulnerability, coping resources, and social context.^1^^,^^2^ A complementary strand of clinical research highlights that vulnerability is not exhausted by average risk alone: studies of depressive relapse and recurrence show that subsequent worsening depends on factors such as prior episode burden, residual symptoms, chronic stress exposure, and limited social support.^17^ Related work on personalized treatment selection has argued that clinically useful prediction should move beyond average effects toward estimating which patients are more likely to benefit from one strategy than another, as illustrated by the personalized advantage index framework.^18^ In practice, stepped-care models reflect the same underlying principle: that monitoring intensity and support level should be matched to predicted individual need rather than applied uniformly.^19^ Our use of uplift modeling is conceptually aligned with this individualized decision logic—rather than estimating only average risk, it seeks to rank differential vulnerability under alternative exposure conditions at the individual level, while remaining appropriately cautious about the observational and proxy-based nature of the treatment and outcome variables in our data.

Building a single model that can generalize treatment-effect rankings across diverse mental-health cohorts remains challenging. Recent work by Bica and van der Schaar^20^ illustrates progress in this direction by proposing a heterogeneous transfer-learning framework for treatment-effect estimation across datasets. Adaptive machine-learning approaches have also shown promise for capturing longitudinal changes in depression and anxiety that static models cannot,^21^ and adaptive data-driven architectures more broadly have been argued to be necessary for addressing the dataset differences inherent in mental-health care.^22^ Theoretical foundations further support this direction: generalization bounds have been established for individualized causal-effect estimators and invariant representation learning has been shown to improve cross-domain causal generalization^23^; neural network-based transfer learning under structured constraints can similarly aid treatment-effect estimation in patient populations not seen during training.^24^ At the same time, cross-dataset generalization remains a well-documented challenge in mental-health applications.^11^^,^^12^ One promising family of methods for learning invariant representations are domain-adversarial neural networks (DANNs), which have shown success in removing domain bias and improving model generality across several health-related settings.^25^^,^^26^^,^^27^ For mental health-related EEG signals, domain adaptation has demonstrated strong cross-subject generalization: Wu et al.^28^ achieved >90% cross-subject accuracy on the SEED dataset by aligning marginal and conditional distributions, and Ju et al.^29^ showed that pseudo-label-based DANN narrows the performance gap between source and target subjects. In neuroimaging, Wang et al.^30^ successfully corrected scanner/site differences across 20 sites for schizophrenia and brain-age prediction using a weighted ERM framework with small amounts of target data, illustrating that adaptation can also be effective in partially supervised settings. However, effectiveness varies across populations and shift mechanisms; for example, unsupervised domain adaptation methods did not outperform simple re-training under temporal dataset shift in a multi-hospital intensive care unit (ICU) study.^31^

Alongside robustness to distribution shift, ensuring fair performance across demographic groups is an important goal in mental-health prediction, since unequal model behavior can reinforce existing disparities in access, diagnosis, and treatment.^14^^,^^32^ Kim et al.^33^ proposed domain-adversarial training to remove gender information from speech features in a PTSD/depression detector, improving both accuracy and fairness across genders. Causal fairness frameworks have similarly revealed that women and Black patients were less likely to receive cardiac therapy than equally eligible peers—a pattern analogous to disparities that can arise in mental-health model outputs.^34^^,^^35^ Cheong et al.^36^ further showed that standard mitigation methods in depression detection reduced but did not fully eliminate gender disparities, suggesting more rigid constraints are needed during training. Applying fairness-aware ideas to uplift modeling can help ensure that differential-vulnerability rankings and downstream support prioritization are not systematically skewed toward majority groups^37^; this integration has seen recent progress in other domains, where causal graphs and rankability constraints have improved treatment-effect estimation in large-scale uplift settings.^38^^,^^39^ Motivated by this literature, we incorporate a simple fairness penalty and domain-adversarial machinery into our uplift model and, given that both fairness interventions and domain adaptation have shown population- and mechanism-dependent effects, we treat them as empirical hypotheses to be evaluated with diagnostics rather than as assumptions of uniform benefit.

The objective of this study is to evaluate whether a single neural uplift model, augmented with domain-adaptation (DA) and fairness-aware components, can produce more reliable treatment-effect rankings across heterogeneous mental-health datasets than a strong non-adapted baseline. Concretely, we ask three questions. First, can a single uplift model accurately rank “who will deteriorate under stress” across three fundamentally different mental-health datasets under leave-one-dataset-out evaluation, and where does this model fail? We use the term *cohort* informally throughout to refer to a distinct study population or dataset, rather than in the technical epidemiological sense of a group followed longitudinally. All three datasets used in this study are cross-sectional. Second, do DA mechanisms such as DANN, conditional adversarial (CDAN), covariance alignment (CORAL), and kernel embedding (MMD) measurably improve out-of-dataset uplift performance relative to a strong shared-encoder baseline without adaptation? Third, how sensitive is cross-dataset uplift to the adversarial alignment weight, and can subgroup uplift gaps remain small while the model still recovers known treatment effects on a semi-synthetic benchmark?

This study makes three contributions. First, we evaluate a unified neural uplift framework with a shared encoder, treatment-specific potential-outcome heads, adversarial domain alignment, and an optional fairness penalty on subgroup uplift gaps. Second, we provide a controlled comparison of several established DA mechanisms under the same encoder and the same leave-one-dataset-out protocol, which lets us assess not only whether adaptation helps, but also where it does not; on average across seeds and folds, none of the evaluated mechanisms achieves a statistically significant uplift gain over the non-adapted baseline, a result that is informative in its own right. And third, we complement performance reporting with a tuning and diagnostic protocol based on adversarial-weight sweeps, scheduling strategies, subgroup-gap audits, and semi-synthetic validation, so that the study contributes practical guidance rather than only point estimates.

**Figure 1:**
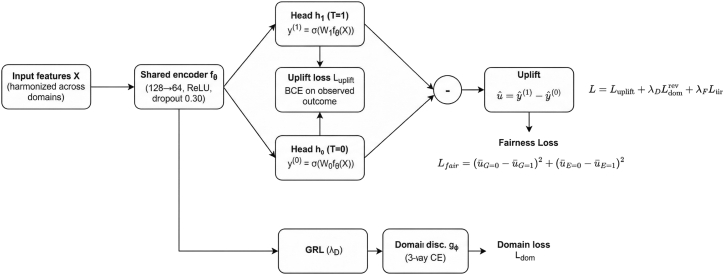
DANN_Uplift architecture and clinical interpretation A shared encoder feeds two potential-outcome heads (*T* = 1/*T* = 0) to produce uplift $\hat{u}$. A gradient-reversal layer routes features to a domain discriminator that encourages domain-invariant representations. An optional fairness term penalizes mean uplift gaps across gender/education subgroups (Equation 10). *Clinical interpretation*, A positive $\hat{u}$ for an individual indicates that, based on the training data, this person is predicted to be more likely to exhibit the adverse outcome if exposed to the treatment proxy than if not. In a prevention context, individuals with high $\hat{u}$ are priority targets for early clinical review; $\hat{u}$ scores should be treated as flags for manual clinical assessment rather than as standalone decision tools. The LODO evaluation protocol approximates the deployment scenario in which a model trained on available study populations is applied to a new population with no labeled outcome data.

Empirically, we study cross-dataset uplift across three study populations: medical students, members of the general public under quarantine, and psychiatric patients. We compare a NoDA against several DA mechanisms under the same backbone, and we additionally examine the effect of a fairness penalty on subgroup uplift gaps. Performance is evaluated primarily with area under uplift curve (AUUC) under leave-one-dataset-out testing, alongside a semi-synthetic benchmark with known treatment effects. This design allows us to distinguish between absolute performance, transfer performance, fairness behavior, and sensitivity to the alignment hyperparameter; the full DANN_Uplift architecture is summarized in Figure 1.

## Results

### Setup and evaluation protocol

We evaluate all models in a leave-one-domain-out (LODO) setting across three study populations: medical students (domain 0, *n* = 881), general population under quarantine (domain 1, *n* = 824), and psychiatric patients (domain 2, *n* = 919); total *n* = 2,624. Dataset-level metadata and treatment/outcome proxy definitions are summarized in Table 1, and the harmonized feature alignment is shown in Table 2. For uplift quality we report area under the uplift curve (AUUC; higher is better), computed on the target (held-out) domain. Unless noted, we use the encoder (128 → 64) with dropout 0.30, Adam, gradient clipping (1.0), and the fairness penalty weight *λ*_*F*_ as indicated per experiment.

**Table 1:** Summary of the three study populations

Study population	*n*	Prev(*T*)	Prev(*Y*)	Treatment proxy *T*	Outcome proxy *Y*
Medical students^40^	881	0.300	0.732	MBI-EX ≥ P75	CES-D ≥ 10
Quarantine (general)^41^	824	0.689	0.498	Growing stress (yes/maybe vs. no)	Coping struggles (yes)
Psychiatric patients^42^	919	0.171	0.285	Anxiety-spectrum dx	Mood disorder/MDD dx

*T* = treatment proxy; *Y* = outcome proxy. Prev(*T*) and Prev(*Y*) denote treatment and outcome prevalence in the final analysis sample. *n* is the final analysis sample after preprocessing and exclusion of non-binary entries in the medical dataset for binary-gender fairness diagnostics.

**Table 2:** Cross-dataset variable alignment after harmonization

Unified feature	Medical	Quarantine	Psychiatric
**Demographics**			

Age	Age (years)	Age (years)	Age (years)
Gender (binary)	Sex (1 = M, 2 = F)	Gender (string)	Sex (string)
Education (years)	12 + MBBS year	— (Imputed)	Education col

**Domain-specific predictors (selected)**			

Trait anxiety	STAI-T	— (Imputed)	— (Imputed)
Burnout (MBI)	MBI-CY, MBI-EA	— (Imputed)	— (Imputed)
Empathy (QCAE)	QCAE-COG, QCAE-AFF	— (Imputed)	— (Imputed)
Stress/coping	— (Imputed)	Frustration, habits	— (Imputed)
Social weakness	— (Imputed)	Social_weakness	— (Imputed)
IQ/cognitive	— (Imputed)	— (Imputed)	IQ score
EEG features	— (Imputed)	— (Imputed)	10 EEG cols

**Treatment and outcome**			

*T*	MBI-EX ≥ P75	Growing stress	Anxiety dx
*Y*	CES-D ≥ 10	Coping struggles	Mood/MDD dx

“—” indicates missing-by-design; such columns are imputed with the training-set median.

### Architecture selection (encoder depth/width)

We first ablated the encoder to choose a stable backbone before any adaptation; the tested configurations and their LODO-AUUC scores are summarized in Table 3.

**Table 3:** Encoder ablation (LODO AUUC)

Encoder	Dropout	Mean AUUC	Per-fold AUUC
128 → 64	0.30	+1.127	(−3.346, +3.309, +3.419)
128 → 64	0.00	+0.666	(−3.125, +2.178, +2.946)
64 → 32	0.20	+0.028	(−2.883, +1.273, +1.693)

Means are across the three held-out domains; parentheses show per-fold AUUC in order (psychiatric, medical, and quarantine).

The (128 → 64) architecture with dropout 0.30 delivered the best average AUUC and was therefore used in subsequent experiments.

### DANN vs. NoDA

**Figure 2:**
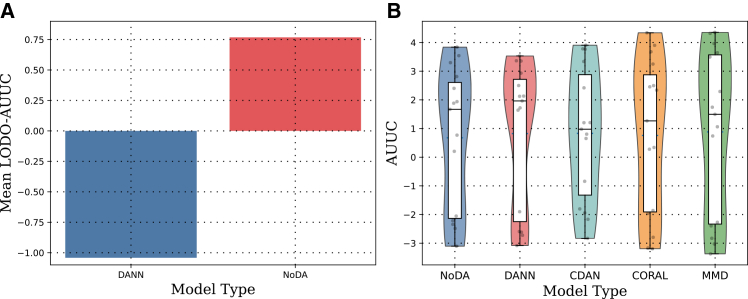
Comparison of uplift performance across modeling strategies (A) Mean LODO-AUUC comparison between DANN and NoDA using a 128 → 64 encoder with dropout 0.30; individual fold scores are overlaid. (B) Distribution of LODO-AUUC scores across five modeling methods (NoDA, DANN, CDAN, CORAL, and MMD); boxplots show IQR and medians, violins show density across 15 seed × fold runs.

With the fixed backbone, we compared DANN training to a NoDA model. The left plot in Figure 2 shows the mean LODO-AUUC comparison between both models using the same seed and preprocessing. In this early comparison DANN and NoDA were close, with a slight edge to NoDA.

Beyond the mean comparison, the right plot in Figure 2 shows the full AUUC distributions across seed × fold samples; the strong overlap anticipates the non-significant head-to-head tests reported later.

### Systematic *λ*_*D*_ analysis and diagnostics

#### Dense *λ*_*D*_ sweep

We ran a grid *λ*_*D*_ ∈ {0.1, 0.2, …, 0.8} with the fixed backbone. Best fold-wise AUUCs were:

$$\begin{array}{lll} & \text{fold }1(\text{psychiatric})=-2.684, & \text{fold}\;2\;(\text{medical})=+4.103,\\ & \text{fold }3(\text{quarantine})=+1.788;\; & \text{mean}=+1.069\pm 2.817. \end{array}$$

**Figure 3:**
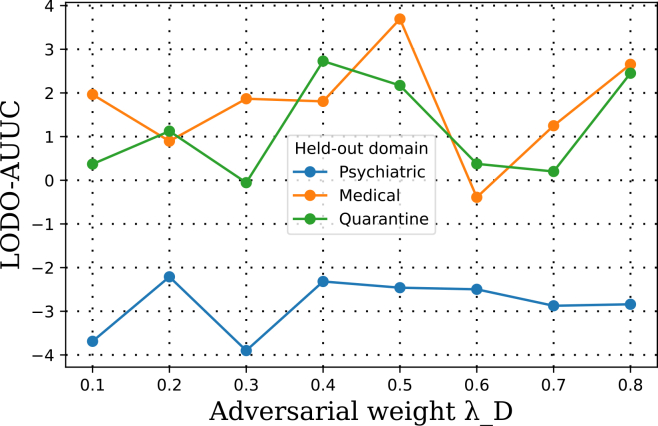
Dense sweep of adversarial weight *λ*_*D*_ for DANN models using a 128 → 64 encoder with dropout 0.30 The *x* axis shows *λ*_*D*_ values from 0.1 to 0.8, and the *y* axis reports LODO-AUUC scores for each fold. Curves represent individual held-out domains (fold 1, psychiatric; fold 2, medical; and fold 3, quarantine). The sweep reveals non-monotonic behavior, with performance gains in folds 2 and 3 and consistent degradation in fold 1, highlighting cohort-specific sensitivity to domain alignment strength.

The sweep revealed a non-monotonic response (Figure 3): performance typically decreased-increased-decreased-increased as *λ*_*D*_ grew, with peaks around 0.7–0.8 for folds 2 and 3, whereas fold 1 stayed negative throughout. This is consistent with trading off removal of harmful domain signal against over-alignment when source/target mechanisms diverge.

#### Fold-wise vs. shared *λ*_*D*_

Selecting *λ*_*D*_ separately per fold improved stability, per-fold AUUC (−2.366, +2.386, +1.883) (mean + 0.634). Using a single shared *λ*_*D*_ (chosen by maximizing mean AUUC in the sweep) gave (−4.485, +2.626, +1.972) (mean + 0.038). The shared setting under-performed due to the psychiatric fold’s sensitivity; this motivated reporting both fold-wise bests and shared settings in later comparisons.

#### Fairness diagnostics

**Figure 4:**
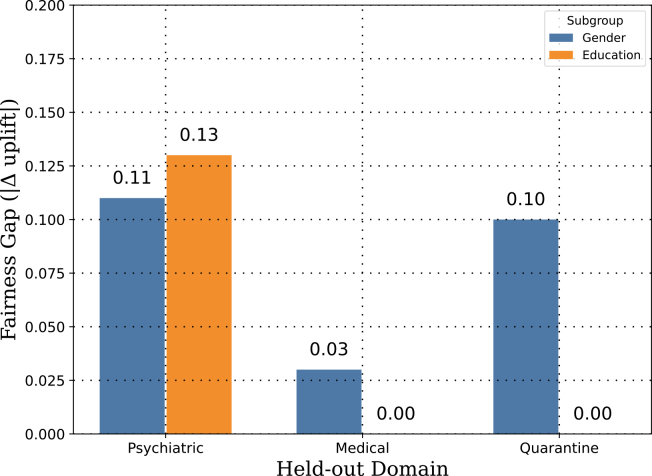
Fairness gaps in uplift predictions across 3-folds (psychiatric, medical, and quarantine) Bars show the absolute difference in mean uplift between gender subgroups and education subgroups within each fold. The *y* axis represents $\left|{\bar{u}}_{\text{group }1}-{\bar{u}}_{\text{group }2}\right|$, a measure of equity in treatment effect estimation. Gaps are small in absolute terms (≈0.00 – 0.13), but given the group-mean parity metric and limited subgroup sizes these figures should be read as a preliminary audit rather than a validated fairness guarantee.

To quantify subgroup equity, we computed absolute uplift gaps by gender and by education among treated samples (Figure 4). Absolute gaps ranged from ≈0.00 to 0.13 across folds, with the largest gap (≈0.13) appearing in the psychiatric cohort’s gender groups. These values are small in absolute terms; however, given the simplicity of the group-mean parity metric, the limited subgroup sample sizes within each fold (particularly in the quarantine cohort where education is missing by design), and the observational nature of the data, we caution against strong conclusions about equity. The fairness figures represent a preliminary audit rather than a validated fairness guarantee; more comprehensive evaluation including intersectional analysis remains future work.

**Figure 5:**
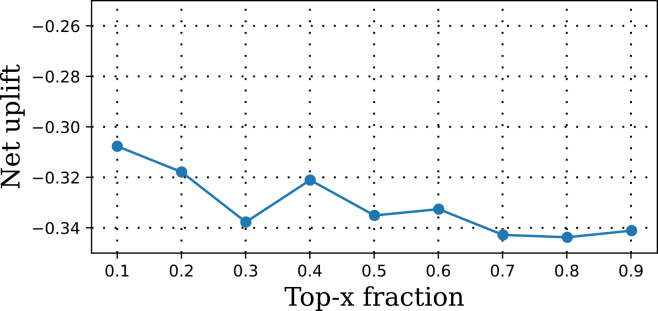
Uplift curve on the psychiatric test fold The curve lies below the random baseline (AUUC ≈−2.66), indicating an inverted treatment-effect ranking. In this fold Pr(*Y* = 1∣*T* = 1) = 0.000 under the present proxy construction: the anxiety-spectrum treatment proxy and the mood-disorder outcome proxy are mutually exclusive diagnostic categories, and both are partially encoded in the predictor features via one-hot encoding of diagnostic-label fields (main.disorder and specific.disorder). The model can learn this negative association from the feature space, producing inverted uplift rankings that reflect proxy-design leakage rather than a DA failure.

Finally, the psychiatric target cohort exhibits a characteristic failure mode: the uplift curve lies consistently below the random baseline, indicating an *inverted* ranking of treatment benefit (Figure 5). This inversion has a precise structural explanation grounded in the proxy design. The treatment proxy (*T* = 1, anxiety-spectrum diagnosis) and the outcome proxy (*Y* = 1, MDD) are derived from the same diagnostic-label columns that are also retained as predictor features after one-hot encoding. In the processed psychiatric dataset, the two proxy categories are mutually exclusive under the present proxy construction: Pr(*Y* = 1∣*T* = 1) = 0.000 compared with Pr(*Y* = 1∣*T* = 0) = 0.344 (Table 4). The model can learn this negative association directly from the feature space, producing negative uplift rankings regardless of domain-alignment strength. The observed negative AUUC therefore reflects *proxy-design leakage* rather than a failure of domain adaptation: when treatment and outcome proxies are derived from overlapping diagnostic categories that are also present as input features, the ranking problem is structurally ill-posed for causal inference. We retain this fold in the evaluation as an instructive case and return to the limitation in limitations and future work subsection.

**Table 4:** Cross-tabulation of treatment proxy *T* and outcome proxy *Y* in the psychiatric dataset (*n* = 919 after harmonization)

	*Y* = 0	*Y* = 1	Total
*T* = 0	500	262	762
*T* = 1	157	0	157
Total	657	262	919
Pr(*Y* = 1)	*T* = 1: 0.000 *T* = 0: 0.344		–

The zero cell (T = 1, Y = 1) confirms perfect mutual exclusivity between the anxiety-spectrum treatment proxy and the mood-disorder outcome proxy under the present proxy construction.

### Schedules, transfers, and semi-synthetic validation

#### Scheduled *λ*_*D*_

We compared fixed *λ*_*D*_ to two schedules (linear ramp and sigmoid ramp). Mean AUUC, *fixed* = +0.557, *linear* = −0.187, and *sigmoid* = +1.327. The sigmoid schedule outperformed fixed and linear, supporting the classical DANN heuristic of delaying stronger adversarial pressure until features stabilize.

#### Single-domain transfers

To isolate cross-domain generalization, we trained DANN on a single source and tested on a single target:

$$\begin{array}{ll} & \text{train 0}\to \text{test }1:+2.815,\;\text{train 0}\to \text{test }2:-2.757,\\ & \text{train }1\to \text{test 0}:+3.291,\;\text{train }1\to \text{test }2:-2.981,\\ & \text{train }2\to \text{test 0}:+2.745,\;\text{train }2\to \text{test }1:+2.003. \end{array}$$

Transfers into the medical domain (0) were consistently positive, while transfers into the psychiatric domain (2) were consistently negative. In light of the proxy audit above, these negative transfers should be interpreted cautiously: they reflect not only cross-domain difficulty but also the structural dependence between psychiatric treatment/outcome proxies and diagnostic input features.

#### Semi-synthetic benchmark (CATE RMSE)

We injected a known treatment effect atop real features to create a sanity-check benchmark. Across two independent runs, fold-wise conditional average treatment effect (CATE) root-mean-square errors (RMSEs) were

[0.264,0.369,0.219](mean 0.284)and[0.238,0.312,0.203](mean 0.251).

These results indicate that, when the data-generating process is well-specified and the injected signal is well-aligned with the model assumptions, the architecture can recover the known effect with low error. Critically, this should be interpreted as a *model behavior check*—a sanity test demonstrating that the architecture does not systematically distort treatment-effect estimates—rather than as evidence of real-world causal identification, which would require assumptions (ignorability, overlap, and stable unit treatment value assumption) that cannot be verified in the observational datasets.

### Head-to-head: DANN vs. CDAN vs. CORAL vs. MMD vs. NoDA

We now report the central comparative evaluation schemes under identical preprocessing, backbone architecture, and LODO evaluation. The compared methods are NoDA, DANN, CDAN, CORAL, and MMD. Each method was subjected to a light grid/Optuna search over (lr, *λ*_*F*_, *λ*_*D*_), with the final search ranges summarized in Table 5, and Table 6 reports the best observed mean AUUC together with uncertainty summaries across 5 seeds × 3-folds.

**Table 5:** Final search ranges for hyperparameters across uplift models

Hyperparameter	Search range	Notes
Learning rate (lr)	1 × 10^−4^–5 × 10^−4^	Log-uniform
Adversarial weight (*λ*_*D*_)	0.1–0.8	Dense grid sweeps; also scheduled ramps
Fairness weight (*λ*_*F*_)	1.0–15.0	Small-to-moderate regularization
Weight decay	10^−5^–10^−4^	Adam

**Table 6:** Summary statistics of LODO-AUUC scores across five modeling methods: NoDA, DANN, CDAN, CORAL, and MMD

Method	Mean	SE	±(95% CI)	sd	*n*
NoDA	+0.678	0.641	±1.255	2.480	15
DANN^43^	+0.823	0.664	±1.300	2.569	15
CDAN^44^	+0.832	0.601	±1.177	2.326	15
CORAL^45^	+0.758	0.705	±1.382	2.731	15
MMD^46^	+0.882	0.752	±1.474	2.913	15

Each method was evaluated over *n* = 15 seed × fold runs. Reported metrics include mean AUUC, standard error (SE), 95% confidence interval half-width, and sample standard deviation. The central finding is that *no* adaptation method achieves a statistically significant improvement over the non-adapted baseline: all pairwise differences are within sampling noise, and the wide standard deviations (*σ* = 2.3 – 2.9 AUUC units) reflect genuine instability under this three-cohort configuration.

The wide standard deviations (*σ* = 2.3–2.9 AUUC units) and confidence-interval half-widths (±1.2–1.5 AUUC) in Table 6 reflect genuine instability in cross-cohort uplift estimation under this sample size and three-cohort configuration. The overlapping distributions visible in Figure 2 reinforce that method choice matters substantially less than dataset characteristics and proxy quality in this setting. These observations should temper any interpretation of point-estimate differences between methods, including the numerical advantage of MMD over NoDA (+0.204 AUUC), which falls well within one standard deviation of either method.

**Figure 6:**
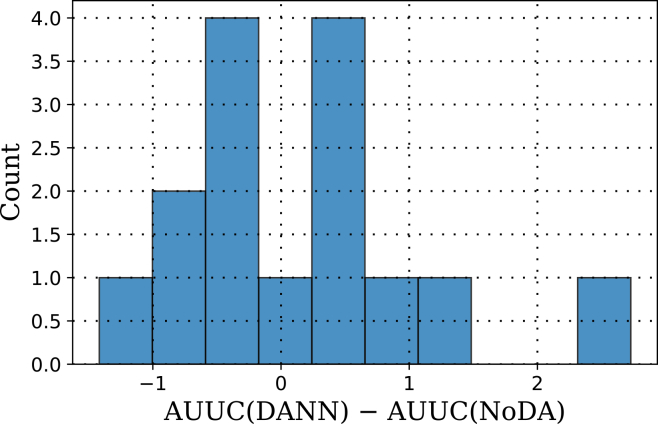
Distribution of paired AUUC differences between DANN and NoDA across seed × fold runs The *x* axis shows AUUC_DANN_ − AUUC_NoDA_ and the *y* axis shows frequency. The distribution is centered near zero (median = 0.064), and the 95% bootstrap confidence interval spans zero, indicating no statistically significant uplift gain from DANN.

Across 5 seeds × 3-folds (*n* = 15 paired samples), the paired median improvement of DANN over NoDA was +0.064 (bootstrap 95% CI [−0.402, 0.511]), with Cliff’s Δ = 0.031 (negligible effect size) and a Wilcoxon signed-rank *p* = 0.847. CDAN vs. DANN and MMD/CORAL vs. DANN were likewise non-significant (all *p* > 0.75). The difference histogram in Figure 6 visualizes the lack of consistent gain.

## Discussion

### Cross-domain uplift performance (RQ1)

Our first question asked whether a *single* causal model can meaningfully rank “who will deteriorate under stress” across multiple cohorts. The LODO experiments indicate that this is feasible but imperfect. In two out of the three unseen test domains (medical students and quarantine respondents), the unified model achieved positive uplift performance (AUUC above zero) on the held-out cohort. For example, when trained on two cohorts and tested on the third, the model obtained relative AUUC gains on the order of +2 to +3 in the medical-student and quarantine cohorts compared with a random-uplift baseline, showing that cross-domain uplift-ranking signal can transfer under some forms of distribution shift.

In practical terms, the model can rank individuals by proxy-defined differential vulnerability in new cohorts better than chance, indicating partial cross-domain generalization at the level of uplift ranking. This addresses RQ1 by showing that a single uplift model can generalize across fundamentally different mental-health datasets, albeit with varying degrees of success. The unified approach also tended to outperform single-source transfers: training on all available source domains generally yielded better uplift ranking in the target domain than training on just one source domain at a time. This suggests that pooling data from multiple cohorts helps the model capture a broader spectrum of stress-outcome relationships, making it more robust than any individual source model. From a practical perspective, the most defensible implication is modest: in a research or service-planning setting, the model output could be used as a *hypothetical triage signal* to prioritize which individuals should receive additional screening or follow-up for stress-related deterioration. It should not be interpreted as a standalone treatment recommendation or as proof that the ranked individuals would truly benefit from a specific intervention in routine clinical practice.

At the same time, the psychiatric cohort exhibited a characteristic failure mode, with the uplift curve lying consistently below the random baseline. As established in systematic *λ*_*D*_ analysis and diagnostics section, this inversion reflects a structural proxy-design issue rather than a DA failure: the treatment proxy (*T* = 1, anxiety-spectrum diagnosis) and the outcome proxy (*Y* = 1, MDD) are derived from diagnostic-label columns that are also retained as input features, and the two proxy categories are empirically mutually exclusive in the processed sample under the current proxy definitions (Pr[*Y* = 1∣*T* = 1] = 0.000; Table 4). The model has access to features that encode this negative association directly, producing inverted uplift rankings regardless of domain-alignment strength. Thus, while RQ1 is answered positively overall, the psychiatric fold illustrates that proxy construct validity is a prerequisite for meaningful uplift estimation: when treatment and outcome proxies are structurally entangled with the input features, the ranking problem is ill-posed for causal inference independent of model choice.

### Value and limits of domain adaptation (RQ2)

RQ2 asked whether adding a DA layer measurably improves out-of-domain uplift performance over a strong non-adapted shared-encoder baseline. The answer is qualified. Across one-shot tuned configurations, the best adversarial methods (DANN and CDAN) achieved numerically higher mean LODO-AUUC than the NoDA baseline (e.g., mean AUUC ≈+0.832 vs. ≈+0.678 on the held-out domains). However, the size of this improvement was small—roughly +0.15 to +0.20 AUUC units—and, given the observed standard errors (≈0.6–0.75) and *n* = 15 paired observations, remained well within sampling noise.

When performance was aggregated across five seeds and 3-folds, the same pattern persisted. DANN and CDAN tended to achieve slightly better point estimates than NoDA, but the paired median improvement of DANN over NoDA was only about +0.06 AUUC, with a 95% bootstrap confidence interval spanning zero and a non-significant Wilcoxon signed-rank test (*p* > 0.8). Comparisons among the adaptation methods were similarly inconclusive: CDAN vs. DANN and CORAL/MMD vs. DANN were also non-significant. Thus, the present evidence does not support a statistically robust advantage of domain adaptation over a strong shared-encoder baseline in this three-dataset setting.

Among the adaptation methods, the adversarial approaches (DANN and CDAN) were numerically strongest, while CORAL and MMD were close behind. This ranking is consistent with the intuition that flexible adversarial aligners may better capture complex cross-domain discrepancies than moment-matching penalties alone. At the same time, the differences among methods were small relative to fold-to-fold and run-to-run variability, so these numerical ordering differences should not be over-interpreted.

The most important conclusion is therefore about magnitude relative to instability. Our results should not be read as showing that domain adaptation is ineffective; rather, they show that any incremental benefit is of comparable size to the noise level under the present sample size and proxy definitions. Similar findings have been reported in other medical domain-shift settings. For example, Guo et al.^31^ found that several domain generalization and adaptation methods did not substantially outperform a strong ERM baseline under temporal dataset shift in ICU data. Our results echo that pattern: when source domains are already diverse and the baseline model is strong, standard DA methods may offer only incremental gains.

Overall, RQ2 is answered cautiously. Domain adaptation may provide modest numerical improvements in cross-dataset uplift ranking, but in the present three-cohort configuration these gains are unstable and not statistically significant. Accordingly, domain adaptation should be treated as an empirical option worth testing, not as a guaranteed improvement.

### Robustness, fairness, and causal validity (RQ3)

RQ3 focused on robustness: how sensitive are our conclusions to the choice and schedule of the adversarial weight *λ*_*D*_, and can subgroup uplift gaps remain modest while the model still recovers known treatment-effect structure on the semi-synthetic benchmark?

#### Tuning *λ*_*D*_ for stable alignment

A key methodological insight is that performance is highly sensitive to *λ*_*D*_. Dense sweeps revealed a non-monotonic relationship between *λ*_*D*_ and AUUC: very small values produced almost no alignment (essentially reducing to NoDA), whereas large values caused over-alignment that degraded performance, likely by forcing the model to ignore meaningful cohort-specific signal. Between these extremes, an intermediate range of *λ*_*D*_ maximized validation AUUC, trading off bias reduction against over-smoothing.

Scheduling *λ*_*D*_ over training further improved stability. In particular, a smoothly increasing sigmoid schedule often matched or exceeded the best fixed setting (e.g., mean AUUC around 1.3 with a ramp vs. roughly 0.6 with a fixed moderate value) and reduced run-to-run variance. This supports the heuristic, seen in original DANN work,^43^ that the adversarial loss should be introduced gradually so that the encoder first learns predictive features and only later enforces domain invariance. Arbitrary static choices of *λ*_*D*_ risk either under-utilizing or over-driving the adversarial signal. Our sweep-plus-schedule protocol turns what is often a trial-and-error process into a reproducible strategy for future studies.

#### Semi-synthetic validation

To probe model behavior under a controlled treatment-effect signal, we injected a known treatment-effect function on top of real features and evaluated CATE error. Across two independent runs, fold-wise CATE RMSEs were (0.264, 0.369, 0.219) (mean 0.284) and (0.238, 0.312, 0.203) (mean 0.251), respectively. These results indicate that when the underlying signal is well aligned with the model assumptions, our architecture can recover a known injected signal with low error under controlled conditions. This makes it more plausible that failures on real datasets reflect proxy-design or construct mismatch rather than a purely architectural problem, although it does not by itself establish causal validity in the observational setting. As noted in uplift target and semi-synthetic ground truth section, this experiment should be read as a model behavior check, not as evidence of real-world causal identification, since the assumptions required for causal inference cannot be verified in the observational datasets.

#### Fairness diagnostics

We quantified subgroup equity by computing absolute uplift gaps between demographic groups among treated individuals (gender and education; Figure 4). Across folds, the gaps were small overall (∼0.00–0.13) with mild variation by domain. Aggregating across seeds and folds using nonparametric summaries (paired bootstrap for the median difference, Cliff’s *δ*), we did not observe a consistent reduction of these gaps attributable to adversarial adaptation: the bootstrap 95% confidence intervals for the gap difference (DANN − NoDA) overlapped zero for both gender and education, and effect sizes were negligible. In other words, on these cohorts the fairness metric remained broadly comparable between DANN and the non-adapted baseline, likely because (i) baseline gaps were already small and (ii) the dominant challenge was label/proxy mismatch, which domain alignment does not directly address.

Overall, RQ3 is answered with a mixed message: careful tuning and scheduling of *λ*_*D*_ are crucial for stable performance; our architecture can recover a known injected signal in a semi-synthetic setting; and fairness gaps are modest and not substantially altered by DA. Robustness and subgroup parity can be partially supported, but they do not automatically follow from adversarial alignment alone.

### Cohort-specific insight: The psychiatric domain

The most striking cohort-specific result was the consistently negative uplift in the Psychiatric test fold (AUUC ≈–2.66; Figure 5). This inversion is not best explained by DA failure alone and is most plausibly explained by the structure of the proxy definitions. As documented in systematic *λ*_*D*_ analysis and diagnostics section and Table 4, the treatment proxy (*T* = 1, anxiety-spectrum diagnosis) and the outcome proxy (*Y* = 1, MDD) are both derived from the diagnostic-label columns main.disorder and specific.disorder, which are also retained as predictor features after one-hot encoding. In the processed psychiatric dataset, these two proxy categories are empirically mutually exclusive in the processed sample under the current proxy definitions: Pr(*Y* = 1∣*T* = 1) = 0.000 versus Pr(*Y* = 1∣*T* = 0) = 0.344 (Table 4). Because the model has access to features that directly encode the proxy relationship between *T* and *Y*, it can learn this negative association from the feature space regardless of alignment strength, producing inverted uplift rankings as a consequence of proxy-design leakage rather than genuine domain-shift failure.

This case illustrates why adversarial domain adaptation cannot rescue a structurally entangled proxy design. Domain-adversarial training targets the marginal feature distribution *P*(*X*), encouraging the encoder to produce representations that are invariant across study populations. However, when the treatment-outcome relationship is already partially inferable from the input features—because both proxies are derived from the same categorical variable—aligning *P*(*X*) across domains changes nothing about the shortcut the model exploits. This is consistent with the broader observation, reported by Guo et al.,^31^ that adversarial methods, CORAL, and MMD all fail to improve generalization when the feature-outcome association in the target domain is fundamentally different from the source: aligning feature distributions cannot correct a misspecified proxy relationship. The psychiatric fold is a concrete clinical instance of this failure mode: the structural dependence between the diagnostic treatment and the diagnostic outcome means that the uplift ranking problem is ill-posed for causal inference in this fold, independent of what alignment method is applied.

The practical lesson is specific and actionable. Before deploying an uplift model in a clinical setting where treatment and outcome are both operationalized from diagnostic categories, practitioners must verify that the treatment proxy is derived from a variable independent of the outcome classification. In the psychiatric context, this would mean using a validated continuous anxiety-symptom scale rather than the primary diagnostic category as the treatment proxy, ensuring that treatment assignment is not structurally entangled with the outcome. More broadly, the psychiatric fold provides a worked diagnostic case: rather than dismissing the negative AUUC as a modeling failure, examining the proxy design revealed the source of the inversion precisely. This kind of structural post-hoc audit—checking Pr(*Y* = 1∣*T* = 1) before interpreting uplift results—should be a routine step in any multi-dataset causal uplift pipeline, particularly when proxies are derived from categorical clinical variables.

### Comparison with existing literature

Qualitative comparison with prior work is summarized in Table 7. We use “TEs” to denote treatment effects, “ITEs” for individual treatment effects, and “DA” for domain adaptation in the comparison table. Transfer CATE methods such as HTCE-learners,^20^ Kyono et al.’s domain-adapted ITE model selection,^47^ and Sun et al.’s cross-domain treatment-effect estimation framework^48^ explicitly target individual treatment effects under distribution shift, but they are not set in mental-health cohorts and do not study fairness or uplift gaps. Conversely, recent fairness-aware uplift approaches (FairUDT,^37^ and fairness evaluation without ground truth^50^) focus on equity in marketing uplift campaigns, without cross-dataset transfer or domain adaptation. Complementary work in mental-health analytics applies domain adaptation to *risk prediction* rather than treatment effects, for example M3BAT for multimodal mobile health sensing^51^ and unsupervised cross-site fMRI adaptation for MDD identification.^52^ These models operate on single outcomes and do not estimate uplift or CATE.

**Table 7:** Qualitative comparison of related work on treatment-effect/uplift modeling, domain adaptation, and fairness

Study	Mental-health outcome	ITE/uplift TE	Multi-dataset/domain	DA for TE/uplift	Fairness in uplift/TE	DA diagnostics
Bica et al.^20^	✕	*✓*	*✓*	*✓*	✕	✕
Kyono et al.^47^	✕	*✓*	*✓*	*✓*	✕	✕
Sun et al.^48^	✕	*✓*	*✓*	*✓*	✕	✕
Shang et al.^49^	✕	*✓*	✕	✕	✕	✕
Zahid et al.^37^	✕	*✓*	✕	✕	*✓*	✕
Kadioglu et al.^50^	✕	*✓*	✕	✕	*✓*	✕
Meegahapola et al.^51^	*✓*	✕	*✓*	✕	✕	*✓*
Fang et al.^52^	*✓*	✕	*✓*	*✓*	✕	✕
Wang et al.^38^	✕	*✓*	✕	✕	✕	✕
He et al.^39^	✕	*✓*	✕	✕	✕	✕
Our study	*✓*	*✓*	*✓*	*✓*	*✓*	*✓*

A tick (*✓*) indicates that the aspect is an explicit, primary focus of the study. Note: Meegahapola et al.,^51^ apply domain adaptation to risk prediction tasks, not to treatment-effect or uplift estimation. The last column (“DA diagnostics”) is marked when the study provides an explicit analysis of domain-adaptation behavior (e.g., sensitivity to alignment strength, failure modes, or ablation of DA components).

Beyond the above categories, a few very recent studies have proposed novel techniques for uplift modeling within a single domain. Wang et al. developed an uplift model that integrates causal inference with graph neural networks to exploit feature relationships, achieving lower error on simulated and real marketing data.^38^ He et al. presented a framework to improve uplift ranking by introducing loss functions that directly penalize mis-ordered treatment effect predictions, demonstrating superior performance in e-commerce targeting scenarios.^39^ While effective in their respective contexts, neither of these approaches incorporates domain adaptation or multi-dataset generalization, and they do not consider fairness constraints. This highlights that the literature has largely treated uplift modeling, domain shift, and fairness as separate problems.

To our knowledge, our study is among the first to jointly combine (i) uplift modeling across three heterogeneous real-world mental-health cohorts, (ii) adversarial representation learning for cross-cohort transfer, and (iii) an explicit fairness regularizer on subgroup uplift gaps together with a systematic analysis of when domain adaptation helps and when its gains over a strong non-adapted baseline are modest. In Table 7, our study is the only one in this comparison to address all six dimensions simultaneously—spanning mental-health outcomes, individualized treatment-effect estimation, multi-dataset generalization, fairness considerations, and diagnostics of DA behavior. Whether this combination yields reliable clinical benefit remains an open empirical question; our findings suggest it is a necessary but not sufficient condition.

### Clinical and public-health implications

The findings carry three practical messages for researchers and practitioners seeking to apply uplift modeling in mental-health settings.

#### Cross-context transferability is feasible but limited

The model achieved positive uplift ranking in two of three held-out populations (medical students and quarantine respondents), suggesting that features predictive of stress-related deterioration in one population may carry signal for another. As noted in cross-domain uplift performance (RQ1) subsection, this should be interpreted modestly: in a research or service-planning context, model output could serve as a *hypothetical triage signal* to prioritize individuals for additional screening or follow-up, not as a standalone clinical recommendation. In deployment, predictions from a model trained on one population and applied to another should be treated as preliminary flags for human clinical review—not as proof that ranked individuals would benefit from a specific intervention in routine practice.

#### Proxy construct validity is a prerequisite, not an afterthought

The psychiatric fold provides the clearest practical warning in this study: when treatment and outcome proxies are derived from overlapping categorical variables that also appear as input features, the uplift ranking problem is structurally ill-posed (Pr(*Y* = 1∣*T* = 1) = 0.000 in the psychiatric fold; Table 4). No unsupervised DA method evaluated here can by itself rescue this configuration. Before deploying an uplift model in a new mental-health context, we recommend verifying three conditions: (i) the treatment proxy is derived from a variable that is *independent* of the outcome classification (e.g., a validated continuous severity scale rather than a diagnostic category); (ii) the direction of the expected treatment-outcome relationship is *a priori* plausible and does not contradict the dataset’s coding structure; and (iii) the treated group has adequate prevalence—our results suggest that very low treatment prevalence (as in the psychiatric fold, Prev[T] = 0.171) can substantially increase instability.

#### Fairness monitoring is necessary, not optional

Demographic uplift gaps in our experiments were small (≈0.00–0.13) and were not substantially altered by adversarial adaptation, suggesting no large subgroup disparities under this group-mean parity metric. However, small gaps under our group-mean parity metric do not constitute clearance for deployment: intersectional disparities (e.g., women with low education vs. men with high education), within-group variance, and allocation-level inequities are not captured.^53^ Clinical AI models should undergo ongoing fairness auditing across all protected attributes available in the target population.^14^ Our fairness penalty is a first step; it does not substitute for regulatory-grade bias evaluation, and any deployment should involve clinicians in reviewing whether predicted uplift gaps align with their ethical and institutional standards.

### Limitations and future work

While our study provides encouraging evidence for domain-adaptive uplift modeling, it also has several limitations that suggest avenues for future work.^54^

First, the magnitude of improvement due to adaptation was small, and we lacked statistical power to definitively assert superiority of our approach over a well-tuned baseline. This is partly due to sample size and the inherent noise in estimating individualized treatment effects. In the future, conducting experiments on larger or additional cohorts would help confirm the trends observed here (e.g., whether adversarial adaptation consistently yields a few percentage points of AUUC gain) or reveal stronger effects. A larger scale study might include more diverse mental health datasets—for example, data from different countries or contexts—which would also test the limits of generalization and possibly amplify the benefits of adaptation (especially if some new domains are substantially different from those seen in training).

Second, the architecture of the model used was relatively simple, as it was a fully connected network with two hidden layers. However, it is possible that using more expressive or deeper encoders could improve performance across domains. Indeed, the recent literature on the topic supports the claim that using more capable or pre-training the model on large datasets can improve the learning of invariant features. For instance, using a transformer-based model pre-trained on large behavioral datasets can improve the learning of universal patterns of stress and coping, thus improving the robustness to domain differences. Exploring such foundation models or multi-modal deep architectures (e.g., incorporating text or wearable sensor data if available) is an exciting direction. However, using deeper models also makes the problem of overfitting more likely and might require careful regularization when data are limited. A related point is that we trained from scratch on these cohorts; using transfer learning from a large corpus (not necessarily mental health-specific, even something such as a general health dataset) could initialize the model in a more domain-invariant space. Future work could evaluate fine-tuning such pre-trained models for uplift predictions, which might yield a stronger baseline and potentially amplify uplift gains or fairness.

Third, our domain adaptation was purely unsupervised, with no target outcome data used during training. In practice, if a small size of labeled target-domain data are available, semi-supervised or few-shot adaptation may yield larger gains than unsupervised alignment alone. A two-stage strategy—first learning a shared representation through adversarial alignment, then fine-tuning the uplift heads on limited target-domain supervision—may be especially useful when the target domain differs in proxy semantics or base rates.

Fourth, although we addressed fairness in terms of parity across two attributes, the ethical and practical aspects of deployment warrant ongoing attention. Our fairness penalty was a simple linear regularizer; future work could investigate richer fairness criteria, such as allocation-level equity or user-defined utility constraints. We also acknowledge that we focused on only two attributes (gender and education); other demographics and their interactions should be considered in a more comprehensive fairness analysis.^55^^,^^56^

Future work should also consider establishing a more systematic benchmark for cross-domain uplift modeling in mental health. Our study is among the first to evaluate multiple domain adaptation techniques (DANN, CDAN, CORAL, and MMD) side-by-side on multiple mental-health cohorts with uplift metrics. Expanding this to more datasets and perhaps standardized tasks could create a benchmark analogous in spirit to WILDS (a benchmark of in‑the‑wild distribution shifts spanning diverse data modalities and applications, from tumor identification to wildlife monitoring to poverty mapping), but for causal uplift scenarios.

#### Construct validity

The fundamental limitation of this study is that the three datasets operationalize “treatment” and “outcome” in qualitatively different ways (Construct validity and comparability section). Even after binarization, AUUC summarizes performance on *proxy-defined* treatment effects, not on true clinical intervention effects. The psychiatric fold provides the sharpest illustration: when treatment and outcome are both derived from a shared categorical variable, the uplift ranking problem is structurally ill-posed regardless of model sophistication. More broadly, a positive AUUC on any fold confirms that the model ranks individuals by their proxy-defined differential vulnerability better than chance—not that those individuals would genuinely benefit from a clinical intervention. Future work should prioritize datasets where the treatment is a real-world intervention measured prospectively with validated instruments, and should also explore domain-specific proxy design, multi-task formulations, or meta-learned sharing mechanisms when a single proxy cannot be meaningfully imposed across all datasets.

#### Gap between semi-synthetic validation and real-world causal inference

The semi-synthetic experiment demonstrates that the architecture can recover a known artificially injected signal with low error (CATE-RMSE ≈0.25–0.28). This does not imply it recovers the true CATE in the observational datasets, where unmeasured confounders—including pre-existing comorbidities, socioeconomic factors, and help-seeking behavior—may bias uplift estimates in unknown directions. Bridging this gap requires either randomized or quasi-randomized data in which treatment assignment is independent of confounders, or a formal sensitivity analysis for unmeasured confounding.^57^ Neither was feasible here given the cross-sectional observational design of all three datasets; we flag this as a prerequisite for any follow-up study that aims to make stronger causal claims.

#### Scalability and adversarial training stability

All experiments were run on a single graphics processing unit (T4, 16 GB VRAM). With larger or more diverse cohorts, adversarial training instability may worsen: the gradient-reversal mechanism can oscillate when the domain discriminator and encoder are unevenly matched in capacity or when batch sizes are small relative to the number of domains. More sophisticated stabilization strategies—such as spectral normalization, Wasserstein-based divergence objectives,^58^ or gradient penalty regularization—may be warranted in larger-scale settings. These extensions were outside the scope of the present study but represent an important direction for making domain-adaptive uplift modeling practically deployable at clinical scale.

Overall, this study provides a careful empirical assessment of cross-dataset uplift modeling in mental-health settings. The results show that uplift-style ranking can transfer across some populations, that domain adaptation may help modestly but not reliably, and that proxy design can dominate model behavior when treatment and outcome are not cleanly separated from the input space. By addressing RQ1-RQ3, the study clarifies both the promise and the current limits of domain-adaptive uplift modeling under heterogeneous proxy definitions. The study also highlights hyperparameter tuning and fairness considerations as important parts of any practical cross-dataset uplift pipeline rather than auxiliary add-ons. For the scientific community, the study’s findings suggest the importance of validating mental health intervention models developed for different cohorts and the possible need for adjustments through domain adaptation, proxy re-evaluation, or fairness constraints—to ensure they remain both effective and equitable.^59^^,^^60^ Going forward, combining adversarial domain-invariant learning with techniques like meta-learning, deeper architectures, and domain-specific knowledge integration holds promise for achieving more robust, scalable, and potentially clinically useful uplift modeling across diverse populations. We hope this study serves as a valuable stepping stone toward that goal, helping to chart a path for future research in generalizable and fair personalized mental health interventions.

## Resource availability

### Lead contact

Requests for further information and resources should be directed to and will be fulfilled by the lead contact, Abdullah Nayem Wasi Emran (abdullah.nayem@bracu.ac.bd).

### Materials availability

This study did not generate new materials.

### Data and code availability

• All datasets analyzed in this study are publicly available from third-party repositories and were not generated by the authors. The Identification of Major Psychiatric Disorders Dataset is available at https://osf.io/8bsvr/. The Medical Students Mental Health Dataset is available at https://zenodo.org/records/5702895. The Mental Health Depression During Quarantine Life Dataset is available at https://data.mendeley.com/datasets/pxjmjyfdh2/1. All datasets are openly accessible subject to the terms of their respective repositories.
• The code generated during this study is publicly available at https://github.com/Nayem9274/domain-adaptive-uplift-mh. The repository contains the analysis notebook and a requirements.txt specifying all software dependencies needed to reproduce the reported experiments.
• No new datasets were generated or deposited as part of this study. This study does not report original data.

## Acknowledgments

This work was conducted at and supported by the 10.13039/501100009500Bangladesh University of Engineering and Technology, Dhaka, Bangladesh.

## Author contributions

A.B.M.A.A.I. and A.N.W.E. jointly developed the research idea. A.N.W.E. was responsible for data collection, coding, and analysis. A.B.M.A.A.I. supervised the work. A.N.W.E. prepared the manuscript, and A.B.M.A.A.I. reviewed and refined it.

## Declaration of interests

The authors declare no competing interests.

## STAR★Methods

### Key resources table

**Table:** 

REAGENT or RESOURCE	SOURCE	IDENTIFIER
**Deposited data**		

Identification of Major Psychiatric Disorders Dataset	Park et al.^42^	https://osf.io/8bsvr/
Medical Students Mental Health Dataset	Carrard et al.^40^	https://zenodo.org/records/5702895
Mental Health Depression During Quarantine Life Dataset	Amin et al.^41^	https://data.mendeley.com/datasets/pxjmjyfdh2/1

**Software and algorithms**		

Python 3.10	Python Software Foundation	https://www.python.org
PyTorch 1.13	Paszke et al.^61^	https://pytorch.org
scikit-learn	Pedregosa et al.^62^	https://scikit-learn.org
Optuna	Akiba et al.^63^	https://optuna.org
Code for this study	This paper	https://github.com/Nayem9274/domain-adaptive-uplift-mh

### Method details

#### Notation and formal definitions

##### Potential outcomes and individual treatment effect

Following the Neyman–Rubin potential-outcomes framework,^64^ let *T* ∈ {0, 1} denote a binary treatment indicator, *Y*(*t*) the potential outcome under treatment value *t*, and $x\in R^{d}$ a vector of pre-treatment covariates. The *Conditional Average Treatment Effect* (CATE) is

(Equation 1) $$CATE\left(x\right)=E\left[Y\left(1\right)-Y\left(0\right)|X=x\right].$$

Because only one potential outcome is observed per subject, CATE cannot be computed directly from observed data alone.

##### Uplift

In uplift modeling,^65^ the model estimates a plug-in approximation of CATE for binary outcomes:

(Equation 2) $$\hat{u}\left(x\right)=\hat{P}\left(Y=1|X=x,\;T=1\right)-\hat{P}\left(Y=1|X=x,\;T=0\right).$$

A positive value indicates that the treatment is predicted to increase the outcome probability for individual **x**; a negative value indicates the reverse. Individuals are ranked by $\hat{u}$ to prioritize those predicted to show the greatest differential vulnerability under the exposure proxy—that is, those most likely to deteriorate under treatment relative to control—who are therefore the primary targets for monitoring or low-intensity support.

##### Area under the uplift curve (AUUC)

The *uplift curve* plots the observed mean treatment effect as progressively larger fractions of the test set are targeted in descending order of $\hat{u}$. At targeting fraction *f*, the net uplift is

(Equation 3) $$NetUplift\left(f\right)=E\left[Y|T=1,\;\hat{u}\geq q_{f}\right]-E\left[Y|T=0,\;\hat{u}\geq q_{f}\right],$$

where *q*_*f*_ is the *f*-th upper quantile of $\hat{u}$. The AUUC is the area between this curve and the random-baseline curve (targeting without uplift information), computed via the trapezoidal rule over ten equal-fraction bins. A *positive* AUUC indicates that the model’s ranking outperforms random selection; a *negative* AUUC indicates an inverted ranking—the model assigns high predicted uplift to individuals who actually benefit less.

##### Leave-one-domain-out (LODO) evaluation

In LODO evaluation, all samples from one study population are held out as the test set; the model is trained on the remaining populations and the held-out population’s unlabeled features are optionally used for unsupervised domain alignment. This protocol approximates the deployment scenario in which a model trained on available populations is applied to a new population with no labeled outcome data.^12^

##### Domain-adversarial training

Domain-Adversarial Neural Networks (DANN^43^) learn a feature representation that is simultaneously predictive of the outcome and invariant with respect to study population membership. A gradient-reversal layer (GRL) multiplies the gradient by −*λ*_*D*_ during backpropagation, causing the encoder to maximize the domain discriminator’s loss while the discriminator itself is optimized normally. The scalar *λ*_*D*_ ≥ 0 controls alignment strength; *λ*_*D*_ = 0 recovers the non-adapted baseline (NoDA).

#### Datasets and problem setup

We use three heterogeneous study populations that differ in population, context, and measurement: medical students (academic stress), a general-population sample collected during COVID-19 quarantine, and a clinical psychiatric sample spanning healthy controls and major disorders. The medical-student sample comprises cross-sectional survey data collected in Switzerland with demographics and psychometrics (e.g., burnout, anxiety, empathy).^40^ The quarantine sample contains population-level survey responses collected in Bangladesh during the COVID-19 lockdown period, with stress-related indicators (e.g., frustration, habit change, mood swings).^41^ The psychiatric sample was collected in South Korea and includes diagnostic labels, cognitive scores, and EEG-derived features.^42^ A descriptive cross-dataset characterization of these three populations—including demographic heterogeneity analyses and cross-context comparisons—is provided in our prior published work.^13^ Table 1 summarizes the key characteristics of each study population; Table 2 maps the available features across datasets.

Data harmonization proceeded as follows. Column names were lowercased, numeric fields were imputed with the median and categorical fields with the mode, and remaining non-numeric columns were one-hot encoded. A single ColumnTransformer fit on the concatenated frame produced a common design matrix $X\in R^{n\times d}$ across domains. Domain IDs *D* ∈ {0, 1, 2} index the medical, quarantine, and psychiatric study populations respectively. For the quarantine dataset, the growing-stress item was originally recorded on a three-level scale (yes/maybe/no); both *yes* and *maybe* responses were mapped to *T* = 1 on the grounds that self-reported uncertainty about increasing stress still reflects a degree of perceived stress elevation, while *no* responses were mapped to *T* = 0. For fairness diagnostics we retain binary *gender* and an *education* bin (e.g., ≥12 years). Five medical students who identified as non-binary (sex = 3) were excluded prior to analysis to ensure valid binary gender encoding for the fairness diagnostic, yielding *n* = 881 retained participants in that sample and a total analytical sample of *n* = 2, 624; the published descriptive analysis of these datasets^13^ reports *n* = 886 and *n* = 2, 629 respectively, as it precedes this exclusion step.

##### Limitations of harmonization

Merging three surveys into a shared feature matrix entails several trade-offs that merit explicit acknowledgment. First, *missing-by-design* columns (Table 2) are imputed with the training-set median, which obscures domain-specific information: for example, the absence of trait-anxiety scores (STAI-T) in the quarantine and psychiatric datasets means the model cannot draw on this clinically important predictor when either of those populations is the test target. Second, one-hot encoding of categorical variables such as occupation in the quarantine dataset inflates feature dimensionality and may introduce spurious correlations that are absent in smaller cohorts. Third, pooled standardization using statistics computed across all three datasets may distort within-population distributions when cohorts differ substantially in means or variances—as is likely between self-report Likert-scale items and the EEG-derived continuous features in the psychiatric dataset. Future work should explore population-specific normalization strategies or modality-specific sub-encoders to mitigate these effects.

#### Construct validity and comparability

The three study populations differ not only in their feature distributions but in the *constructs* that the treatment and outcome proxies represent—a recognized challenge in multi-site machine-learning research.^66^

##### Measurement heterogeneity

In the medical-student sample, the treatment proxy (burnout exhaustion at or above the 75th percentile) is a validated psychometric score derived from the Maslach Burnout Inventory,^67^ offering relatively high measurement reliability. In the quarantine sample, the treatment proxy (growing stress) is a single dichotomous self-report item, which carries greater measurement noise and may conflate stress appraisal with stress exposure^68^—a distinction that matters for causal identification. In the psychiatric sample, the treatment proxy is a clinical diagnosis—a qualitatively different type of variable that reflects a clinician-assigned category rather than a continuous self-report. These differences in proxy type and reliability limit the degree to which treatment effects are directly comparable across study populations.

##### Mitigation strategies

To partially address construct heterogeneity, we adopt three practices. First, all treatment and outcome variables are binarized to a common {0, 1} scale, reducing measurement-scale differences. Second, the semi-synthetic benchmark (Uplift target and semi-synthetic ground truth section) injects a known treatment-effect signal on top of real features, allowing model behavior to be evaluated independently of proxy quality. Third, cross-dataset AUUC comparisons are treated as *ordinal rankings* of differential vulnerability rather than as absolute causal effect estimates.

##### Interpretation caveat

A positive AUUC in this study means that the model ranks individuals by *proxy-defined* differential vulnerability better than chance—it does not mean the model identifies individuals who would benefit from a real-world clinical intervention. Causal identification requires the assumptions of ignorability (no unmeasured confounding), overlap, and SUTVA (Stable Unit Treatment Value Assumption), none of which can be verified in these observational datasets. In the psychiatric dataset a further structural issue applies. The treatment proxy (*T* = 1: anxiety-spectrum diagnosis) and the outcome proxy (*Y* = 1: mood disorder or MDD) are both derived from the same diagnostic-label columns (main.disorder and specific.disorder), which are also retained as predictor features after one-hot encoding. In the processed dataset, the two proxy categories are mutually exclusive under the present proxy construction: Pr(*Y* = 1∣*T* = 1) = 0.000 versus Pr(*Y* = 1∣*T* = 0) = 0.344. The model therefore has access to features that strongly encode the proxy relationship between *T* and *Y*, creating a structural shortcut that conflates proxy-label leakage with causal signal. We flag this as a proxy-design limitation specific to the psychiatric fold and return to it in Systematic *λ*_*D*_ analysis and diagnostics section and Limitations and Future Work subsection.

#### Uplift target and semi-synthetic ground truth

To evaluate individual treatment effects (ITE) under controlled conditions, we construct a semi-synthetic outcome. Each subject is assigned a Bernoulli treatment *T* ∈ {0, 1}, and the observed binary outcome *Y* ∈ {0, 1} is sampled from a response surface that embeds a known ITE signal. This preserves the empirical feature distribution while providing a reference conditional average treatment effect

(Equation 4) $$u^{⋆}\left(x\right)=E\left[Y|X=x,\;T=1\right]-E\left[Y|X=x,\;T=0\right].$$

Critically, this benchmark should be interpreted as a *model behavior check*: it tests whether the architecture can recover a known injected signal without systematic distortion, not whether the model achieves real-world causal identification. Ground-truth ITEs are unavailable in the observational datasets, and the assumptions required for causal identification (ignorability, overlap, SUTVA) cannot be verified in those datasets; we discuss this gap explicitly in Limitations and Future Work subsection. We report ranking-based uplift metrics on the observed outcomes (AUUC) and CATE error (RMSE) on the semi-synthetic benchmark.

#### Model: DANN_Uplift (domain-adversarial uplift network)

Our model comprises three components: a shared feature extractor $f_{\theta }:R^{d}\to R^{m}$, two treatment-specific heads $h_{1},h_{0}:R^{m}\to \left(0,1\right)$ that predict potential outcomes, and a domain discriminator $g_{\phi }:R^{m}\to \Delta^{K-1}$ connected via a Gradient Reversal Layer (GRL).

##### Encoder and potential-outcome heads

Unless stated otherwise, *f*_*θ*_ is a two-layer MLP (128 →  64, ReLU) with dropout 0.30 after the first hidden layer. The potential-outcome heads are single linear layers with sigmoid activations:

(Equation 5) $${\hat{y}}^{\left(1\right)}=h_{1}\left(f_{\theta }\left(x\right)\right),$$

(Equation 6) $${\hat{y}}^{\left(0\right)}=h_{0}\left(f_{\theta }\left(x\right)\right),$$

(Equation 7) $$\hat{u}\left(x\right)={\hat{y}}^{\left(1\right)}-{\hat{y}}^{\left(0\right)}.$$

For an observed tuple (*x*, *t*, *y*) the supervised loss is computed only on the exposed head:

(Equation 8) $$L_{\text{uplift}}=BCE\left(\underset{{\hat{y}}_{\text{obs}}}{\underset{︸}{t\cdot {\hat{y}}^{\left(1\right)}+\left(1-t\right)\cdot {\hat{y}}^{\left(0\right)}}},\;y\right).$$

##### Adversarial domain alignment and fairness regularizer

The discriminator receives features passed through a GRL and predicts the domain label *d*. Let $L_{\text{dom}}$ denote the cross-entropy domain loss; the overall objective is

(Equation 9) $$L=L_{\text{uplift}}+\lambda_{D}\;L_{\text{dom}}^{\left(rev\right)}+\lambda_{F}\;L_{\text{fair}},$$

where $L_{\text{dom}}^{\left(rev\right)}$ indicates the domain loss backpropagated through the GRL (i.e., maximizing domain confusion). The fairness term penalizes disparities in estimated uplift between protected subgroups. For uplift vector $u={\hat{y}}^{\left(1\right)}-{\hat{y}}^{\left(0\right)}$ and binary masks *G* ∈ {0, 1} (gender) and *E* ∈ {0, 1} (education),

(Equation 10) $$L_{\text{fair}}={\left({\bar{u}}_{G=0}-{\bar{u}}_{G=1}\right)}^{2}+{\left({\bar{u}}_{E=0}-{\bar{u}}_{E=1}\right)}^{2},$$

evaluated only when both subgroup cells are present in the minibatch or, for stability, aggregated at epoch level.

##### Scope and limitations of the fairness metric

The fairness penalty in Equation 10 captures *group-mean uplift parity*—a first-order check that the average predicted benefit does not systematically differ between demographic groups. This is a necessary but not sufficient condition for equitable treatment allocation. The metric does not capture: (i) *intersectional* disparities (e.g., women with low education versus men with high education); (ii) distributional inequities within groups, such as differences in the variance or tail behavior of uplift scores rather than only the mean; or (iii) downstream allocation fairness, which depends on how ranked uplift scores are actually used by decision-makers.^69^ We treat the current metric as a preliminary audit and note that more comprehensive fairness evaluation—including intersectional analysis and allocation-level metrics—is an important direction for future work; we return to this in Limitations and Future Work subsection.

#### Baselines and variants

All variants use the same encoder architecture (128–64, dropout 0.30). The compared methods are.

• **NoDA**: no domain alignment (*λ*_*D*_ = 0), standard two-head uplift network.
• **DANN**^43^: domain-adversarial alignment with tuned fixed or scheduled *λ*_*D*_(*p*).
• **CDAN**^44^: conditional adversarial alignment where the discriminator conditions on predictions by concatenating $\left[f\left(x\right),{\hat{y}}^{\left(0\right)},{\hat{y}}^{\left(1\right)}\right]$.
• **CORAL**^45^: non-adversarial covariance alignment that penalizes covariance mismatch between domain embeddings.
• **MMD**^46^: kernel mean embedding alignment (MMD) between source and target embeddings.

All methods use Adam with weight decay in [10^−5^, 10^−4^], gradient clipping at 1.0, and early stopping on validation AUUC. As shown in Figure 1, all methods use a common shared encoder backbone; what differs is the alignment mechanism, allowing a controlled comparison under identical conditions.

#### Evaluation protocol

We evaluate two transfer settings.

1. **Single-source transfer:** train on a single labeled source domain, adapt using unlabelled target batches, and evaluate on held-out target labels.
2. **Leave-one-domain-out (LODO):** train on two domains and test on the third, rotating across folds.

The primary metric is AUUC (area under the uplift curve) on the target; we also plot uplift curves. On the semi-synthetic benchmark we compute CATE RMSE between $\hat{u}\left(x\right)$ and *u*^⋆^(*x*). Fairness is summarized by absolute uplift gaps $\left|{\bar{u}}_{G=0}-{\bar{u}}_{G=1}\right|$ and $\left|{\bar{u}}_{E=0}-{\bar{u}}_{E=1}\right|$ computed on treated subsets. Methods are compared using paired statistical tests on fold-wise AUUC (nonparametric Wilcoxon signed-rank tests with bootstrap confidence intervals and Cliff’s *δ* where applicable).

#### Experimental details

##### Encoder selection

We evaluated several backbones (32–16, 64–32, 128–64) with dropout choices {0.0, 0.30}; the 128–64 backbone with dropout 0.30 yielded the most stable positive mean AUUC under LODO and was adopted as default.

##### Adversarial weight protocol

To ensure reproducibility we followed a systematic protocol for selecting *λ*_*D*_: (i) dense grid sweeps of *λ*_*D*_ over a plausible interval to visualize AUUC sensitivity per fold; (ii) selection of per-fold optimal *λ*_*D*_ and a shared *λ*_*D*_ that maximizes mean AUUC; (iii) evaluation of scheduled ramps (linear and sigmoid) as principled alternatives to fixed values.

##### Diagnostics

The psychiatric study population was the most fragile target: large *λ*_*D*_ values often led to negative AUUC (uplift inversion). However, subsequent proxy checks showed that this inversion is not attributable to domain adaptation alone. In the psychiatric dataset, the treatment and outcome proxies are derived from diagnostic-label fields that are also retained as input features, creating a structurally negative proxy relationship that can induce shortcut learning. We therefore interpret the psychiatric fold as an informative stress test of proxy design rather than as a clean measure of adaptation failure.

##### Hyperparameter search and reporting

We varied the adversarial weight *λ*_*D*_, the fairness penalty weight *λ*_*F*_, and the learning rate (lr), among others. Specifically, *λ*_*D*_ was swept across a dense grid (0.1, 0.2, …, 0.8), and both linear and sigmoid schedules for *λ*_*D*_ were tested. The fairness weight *λ*_*F*_ was also varied via grid search, and we tried several learning rates. The hyperparameter search ranges used across all domain adaptation methods are summarized in Table 5.

### Quantification and statistical analysis

The primary performance metric is the area under the uplift curve (AUUC), computed via the trapezoidal rule over ten equal targeting fractions on the held-out domain under leave-one-domain-out (LODO) evaluation. A positive AUUC indicates that the model’s uplift ranking outperforms random targeting; a negative AUUC indicates an inverted ranking. On the semi-synthetic benchmark, model accuracy is quantified by CATE root-mean-square error (RMSE) between the predicted uplift $\hat{u}\left(x\right)$ and the known injected treatment effect *u*^⋆^(*x*).

All adaptation methods were evaluated across 5 random seeds × 3 held-out folds, yielding *n* = 15 paired seed×fold observations per method comparison. Pairwise method comparisons use the two-sided Wilcoxon signed-rank test on fold-wise AUUC. Effect sizes are reported as Cliff’s *δ*. Bootstrap 95% confidence intervals (1,000 resamples) are reported for the median paired difference. No corrections for multiple comparisons were applied given the exploratory nature of the comparisons; all *p*-values and confidence intervals are reported as-is. The threshold for statistical significance was set at *α* = 0.05; no comparison met this threshold.

Fairness is summarized by the absolute mean uplift gap between demographic subgroups (gender; education) among treated individuals within each fold. No formal significance testing was applied to fairness gaps given the small subgroup sample sizes; these values are reported as descriptive audit statistics.

Full statistical details—including exact *n* per fold, AUUC values per seed and fold, confidence interval bounds, Wilcoxon *p*-values, Cliff’s *δ*, and CATE-RMSE values across runs—are reported in the Results section and Tables.
